# Multifocal Epithelial Hyperplasia: Persistence in Adulthood and Treatment—A Case Report

**DOI:** 10.1155/crid/9974122

**Published:** 2026-05-27

**Authors:** Daniela Lourdes Argueta-Constanza, Lisset Margarita López-Serrano, Florence Juana María Cuadra-Zelaya

**Affiliations:** ^1^ Department of Preventive Dentistry, School of Dentistry, University of El Salvador, San Salvador, El Salvador; ^2^ Department of Pathology, School of Dentistry, University of El Salvador, San Salvador, El Salvador

**Keywords:** case report, HPV, imiquimod, multifocal epithelial hyperplasia, persistence

## Abstract

**Background:**

Multifocal epithelial hyperplasia (MEH) is a rare, benign epithelial pathology associated with the human papillomavirus (HPV) infection, affecting the oral mucosa. It is primarily observed in children, particularly those from indigenous populations of Latin American origin and typically resolves during adolescence. However, this case report presents a patient with persistent lesions into adulthood. Despite pharmacological treatment, no significant improvements were observed.

**Case Report:**

A 55‐year‐old female patient of indigenous descent presented with multiple asymptomatic papillomatous lesions of varying sizes, which had been present since the age of 12. There were no systemic comorbidities. The presence of multiple lesions and the patient′s age, differential diagnoses such as Cowden syndrome and oral papillomatosis were considered and subsequently ruled out following clinical evaluation. Histopathological analysis confirmed the initial findings, establishing a definite diagnosis of MEH. The patient underwent combined surgical and pharmacological treatment, involving the application of 80% trichloroacetic acid and 5% imiquimod.

**Conclusion:**

Few case reports of MEH in adult patients exist, and none have thoroughly evaluated the effectiveness of pharmacological treatments. This case underscores the inconsistency of pharmacological therapies in adults, as most studies have focused predominantly on pediatric populations. Therefore, further investigation into alternative pharmacological approaches is warranted for the management of MEH in adult patients.

## 1. Introduction

Multifocal epithelial hyperplasia (MEH), also known as Heck′s disease, is a rare benign pathology affecting the oral mucosa, including the lips, buccal mucosa, tongue, and palate [1]. The etiology of MEH has been strongly associated with human papillomavirus (HPV), particularly the low‐risk Genotypes 13 and 32, with evidence suggesting a genetic and ethnic predisposition [1–6]. Additional risk factors warranting evaluation include low socioeconomic status, poor oral hygiene, malnutrition, and overcrowded living conditions [2, 6]. Lesions may be exacerbated by masticatory trauma, potentially impairing feeding and altering the patient′s appearance [5]. MEH is generally considered a self‐limiting condition. The presence of HPV in saliva suggests a possible transmission route within families, facilitated by close contact and shared use of kitchen utensils, toothbrushes, and personal hygiene items; however, the exact transmission dynamics remain unclear [1, 5].

MEH is most commonly observed in children and adolescents [1, 5]; but it may affect individuals ranging from 3 to 69 years of age, with a significantly higher prevalence in the first decade of life [2, 6, 7], which has been attributed to the immature immune system in children [5]. The majority of reported cases occur in females [1, 2, 5, 6]. In pediatric patients, a conservative “wait and watch” approach is generally recommended, as the condition often resolves spontaneously. Treatment options in some cases include vitamin supplementation, topical agents such as imiquimod [3], interferons, and trichloroacetic acid (Harris [8, 9]). Surgical intervention is advised for large lesions that compromise function or aesthetics [1, 5]. Although effective treatment protocols have been almost exclusively documented in pediatric populations, similar outcomes have not been consistently observed in adult patients. This clinical case report is aimed at presenting the persistence of MEH into adulthood and its response to combined surgical and pharmacological management. The case description follows the CARE guidelines.

This clinical case describes a severe presentation of MEH, characterized by the extensive number and distribution of lesions throughout the oral cavity, involving broad areas of the buccal mucosa, tongue, labial mucosa, and labial commissures in a patient over 50 years of age. Following referral to the Oral Pathology Clinic at the University of El Salvador′s School of Dentistry and due to the persistent and progressive nature of the condition, the patient underwent multiple surgical interventions over the years, with recurrence manifesting as new lesions at previously treated sites. Pharmacological therapy was later implemented, resulting in partial improvement of lesions located at the labial commissures, whereas no significant response was observed in the remaining oral mucosa.

The pharmacological approaches employed have not been previously reported in cases of comparable severity, nor in adult patients, highlighting the clinical relevance of the present case.

## 2. Case Report

A 55‐year‐old female of indigenous descent was referred to the Oral Pathology Clinic at the University of El Salvador′s School of Dentistry. Her chief complaint involved multiple painless intraoral masses that had been present since age 12. These lesions gradually increased in number and size over several decades, eventually resulting in functional difficulties with chewing and speech. The patient reported no history of prior therapeutic management for the oral masses.

**Figure 1:**
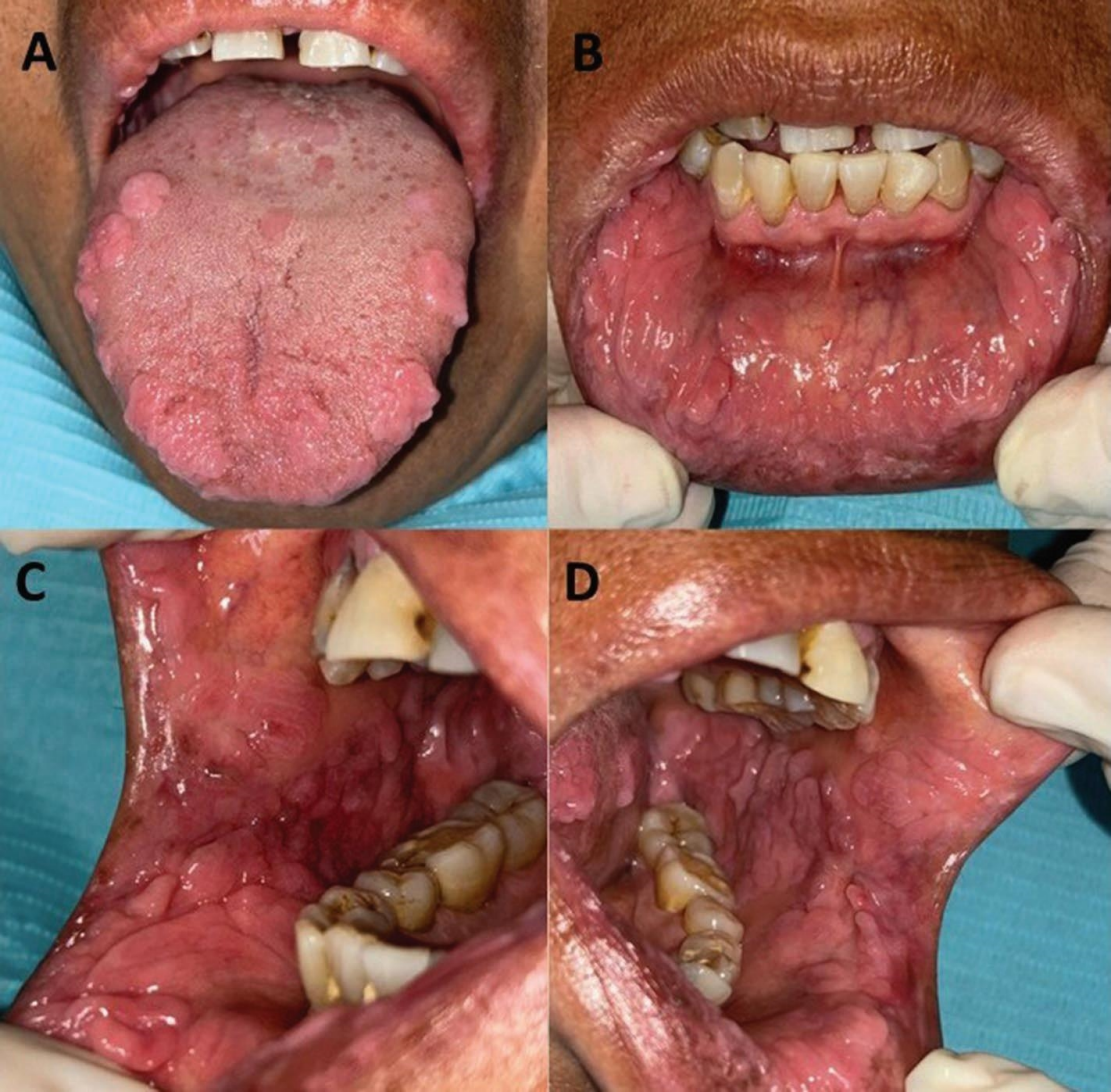
(A)Multiple well‐defined papulonodular lesions were observed on the dorsal surface and margins of the tongue, (B) in the labial mucosa of the lower lip, and (C, D) oral mucosa of the right and left cheeks. The vestibule and attached gingiva appeared free of lesions. The clinical photograph was taken in 2024.

Prior to the clinical evaluation, written informed consent was obtained for the documentation and publication of the clinical findings. Intraoral clinical examination was performed, revealing multiple papillomatous growths of varying sizes bilaterally on the commissural lips, buccal mucosa, lower labial mucosa, and lateral borders of the tongue. The lesions exhibited a “cobblestone” surface, soft consistency, and were asymptomatic upon palpation (Figure 1). No similar lesions were observed elsewhere on the patient′s body. Based on these findings, a clinical diagnosis compatible with MEH was established.

The patient remained systemically healthy throughout the course of care, laboratory tests including complete blood count, coagulation profile, and blood glucose testing were performed and all results were within normal reference limits. Serological testing for human immunodeficiency virus (HIV) was likewise conducted, yielding a negative (nonreactive) result. The patient reported no current use of medications, either prescribed or self‐administered, and denied the use of tobacco, alcoholic beverages, or other illicit substances. No relevant family medical history associated with the condition was reported.

**Figure 2:**
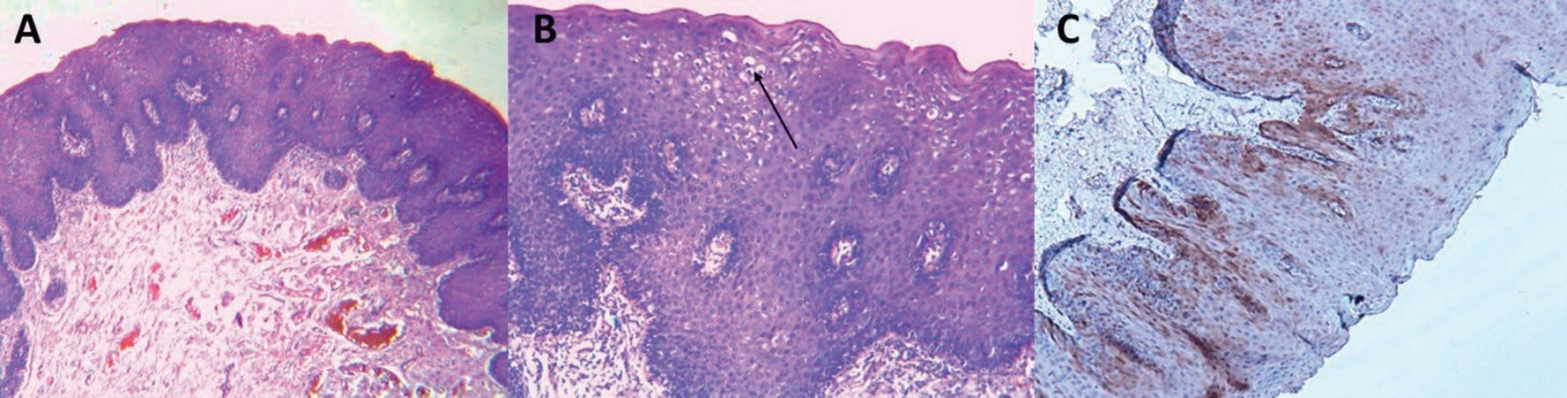
(A) Proliferation of parakeratinized stratified squamous epithelium with acanthosis and broad epithelial rete ridges. (B) Stratified squamous epithelium with acanthosis, abundant keratinocytes displaying pyknotic nuclei with a clear halo, compatible with koilocytes in the spinous layer (black arrow). (C) Immunohistochemical analysis demonstrated less than 70% nuclear and cytoplasmic expression in the epithelial cells.

Treatment consisted of two main approaches based on clinical presentation and lesion‐associated symptoms. The first approach was surgical, involving excisional biopsy of larger lesions interfering with mastication. These lesions, due to their size and location, caused constant discomfort, often aggravated by repeated trauma from contact with teeth and food. Surgical excision was deemed the most appropriate to alleviate symptoms and prevent chronic irritation or secondary complications. Histopathological analysis demonstrated proliferation of stratified squamous parakeratinized epithelium with acanthosis, focal exocytosis, broad epithelial rete ridges, and keratinocytes in the spinous layer showing pyknotic nuclei surrounded by clear halos, consistent with koilocytosis (Figure 2A,B). Subsequently, immunohistochemistry for the p16 marker was performed, yielding a negative result, as less than 30% of the epithelial cells showed nuclear and cytoplasmic positivity (Figure 2C).

**Figure 3:**
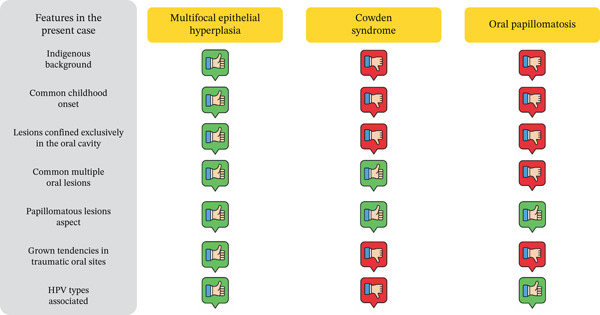
Clinical and histopathological characteristics of the present case compared with its differential diagnoses.

Clinical and histopathological findings confirmed the diagnosis of MEH. The presence of multiple oral lesions, differential diagnoses including systemic and local conditions such as Cowden syndrome—characterized by multiple hamartomatous lesions [10]—and oral papillomatosis, associated with immunosuppression or idiopathic origins [11], were excluded. For definitive diagnosis, the clinical and histopathological features presented by the patient were compared with those of differential diagnoses (Figure 3).

**Figure 4:**
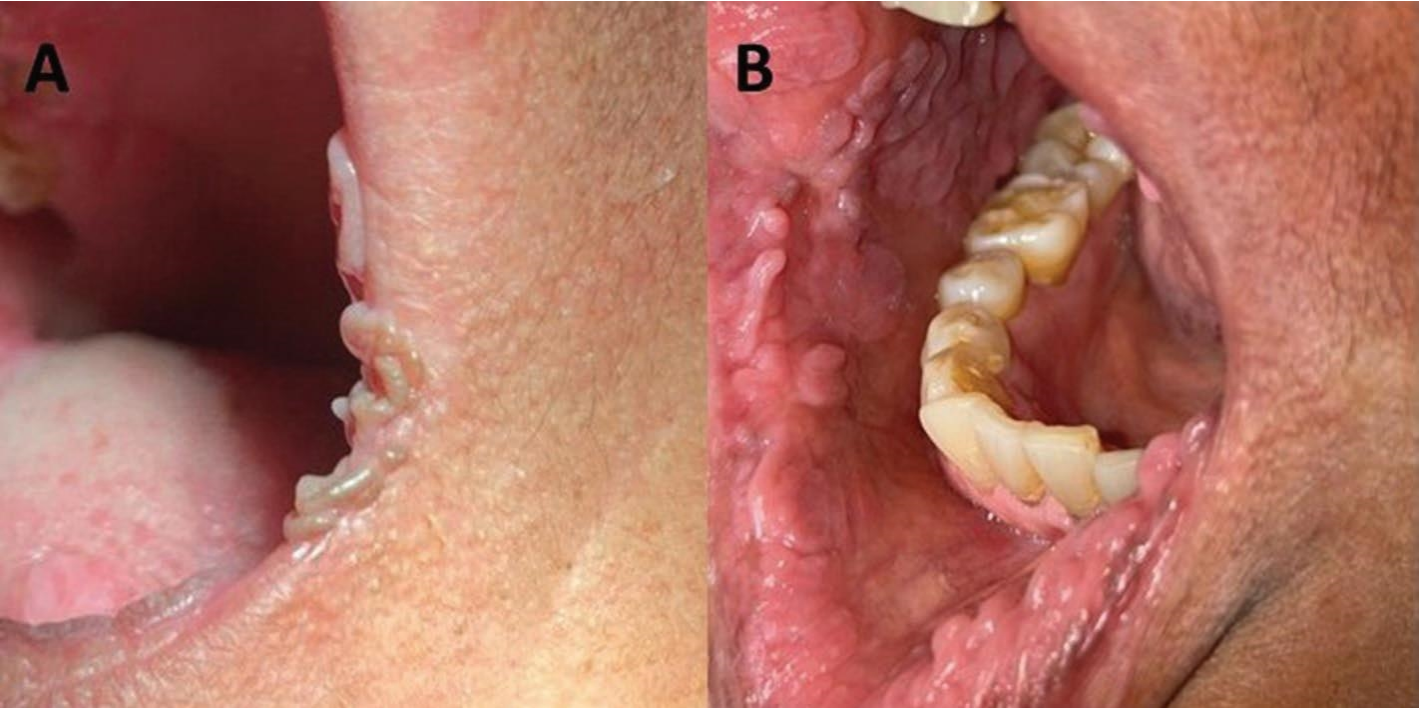
(A) Elongated papulonodular lesions were located bilaterally at the lip commissures, were similar in color to the mucosa, were asymptomatic, and were present prior to treatment with 5% imiquimod. (B) The lip commissures showed no papulonodular lesions after pharmacological treatment with imiquimod. Clinical photographs (A) and (B) were taken in 2023 and 2024, respectively

The second treatment approach was pharmacological, targeting multiple oral lesions that, although asymptomatic, posed significant aesthetic concerns for the patient. Treatment was initiated with 5% imiquimod cream [3, 4] and was applied topically to lesions at the labial commissure and several intraoral sites by placing approximately 0.5 cm of cream on the tip of a sanitized finger and applying it daily at night to the left commissural lip lesions. After 4 months, lesions on the commissural lips were resolved (Figure 4). However, no significant clinical improvement was observed in the lesions affecting the cheeks and lower labial mucosa. Subsequently, 5% imiquimod ([4]) treatment was extended to persistent lesions on the oral mucosa. Adherence to 5% imiquimod therapy was monitored on a weekly basis through patient interviews and visual verification of the 5% imiquimod cream tube.

A total of 80% trichloroacetic acid was administered weekly to smaller intraoral lesions unresponsive to imiquimod using a cotton swab, with application times ranging from 15 s to 1 min depending on patient tolerance of the burning sensation, followed by water rinsing, a protocol similar to that described in other studies ([8, 9]).

**Figure 5:**
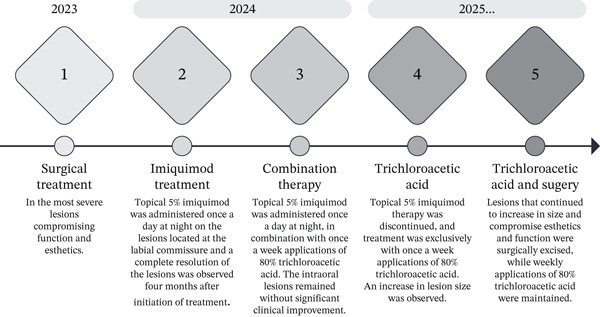
Timeline of surgical and pharmacological treatments in the present case.

The patient reported an overall favorable perception of the treatment and expressed satisfaction with the therapeutic intervention. She described intermittent subjective clinical improvement. A 27‐month period of observation and management was documented, encompassing multiple surgical procedures and pharmacological therapies (Figure 5); no significant changes were observed in the size or number of intraoral lesions. Considering the progressive nature of the lesions and their limited response to pharmacological therapy, the prognosis remains guarded.

## 3. Discussion

The initial cases of MEH were identified among Native populations in the United States, Mexico, Guatemala, and India. In El Salvador, MEH was first reported in 1965, affecting 16.2% of Ladino children in urban areas and 43.4% in rural settings, within an age range of 5–15 years [12]. Currently, MEH cases have been documented worldwide, with evidence suggesting an association between ethnicity and susceptibility to the disease [5]. An epidemiological study conducted in Mexico, with an ethnically similar population to El Salvador, reported a prevalence of 32.3% among children of the Nahuatl population [13]. In the Maya population of southern Mexico, approximately 7% of children around 7 years old presented oral lesions consistent with MEH, with an overall prevalence of 0.1% [14].

Reports of MEH in adults remain scarce, with few documented cases demonstrating persistence of lesions since childhood or the therapeutic approach in this population, as exemplified by the present case. The pathophysiological mechanisms underlying the persistence of lesions into adulthood remain unclear. Given the lesion size, severity (in numbers), and persistence of MEH into adulthood observed in this case, a literature review was conducted to compare it with other similar diseases presenting with HPV‐associated papillomatous lesions and genetic alterations, such as Cowden syndrome (Table 1).

**Table 1:** Differential diagnosis between multifocal epithelial hyperplasia, Cowden syndrome, and oral papillomatosis.

Features	Multifocal epithelial hyperplasia	Cowden syndrome	Oral papillomatosis (papilloma, verruga, and condyloma)
Oral clinical appearance	Multiple, small (2–10 mm), soft, sessile papules or nodules; mucosal‐colored to pale white; often flattened or cobblestone‐like when clustered.	Features: multiple oral papillomas and mucocutaneous papules that can produce a cobblestone.	Solitary exophytic, pedunculated or sessile lesion with cauliflower/filiform surface; usually small (< 1 cm).
Common intraoral sites	Buccal and labial mucosa most common; tongue and gingiva can be involved.	It usually presents on the tongue, buccal mucosa, palate, lips, and gingiva.	Palate, tongue, labial mucosa, and gingiva.
Lesions in other sites	Not applicable.	Breast, thyroid, gastrointestinal, genitourinary, and cutaneous.	Anogenitals and skin.
Usual HPV types/etiology	Strongly associated with HPV 13 and 32 in most series.	It is not associated with HPV.	Commonly HPV 6 and 11 (low‐risk types) in many lesions.
Genetic mutation	Not applicable.	PTEN.	Not applicable.
Histopathologic features	Marked acanthosis with broad rete ridges, hyperparakeratosis; “mitosoid” (atypical) keratinocytes are characteristic; koilocytosis may be minimal or absent.	The most distinctive finding is the trichilemmoma, which shows clear cells rich in glycogen surrounded by a thick basement membrane.	Papillomatous fronds of hyperplastic squamous epithelium with fibrovascular cores; koilocytosis may be present.
Typical age	Mostly children/adolescents; familial clusters reported; higher prevalence in some indigenous populations.	It commonly presents in early adulthood.	Any age but often adults.

*Note: Source:* Author′s analysis of Conde‐Ferráez and González‐Losa [1], Sethi et al. [5], Pîrlog et al. [10], and Andrei et al. [11].

Management of MEH lesions may involve pharmacological and surgical strategies; however, most cases show spontaneous involution. In the present case, a combination of both approaches was implemented. Initially, the patient underwent surgical procedures for the lesions that caused aesthetic and functional problems during chewing and speech. Surgical procedures were not considered viable for the remaining lesions, which were scattered across multiple oral structures, subsequently beginning pharmacological treatments.

Imiquimod is an immune response modifier that has been indicated as a valuable agent in treating anogenital warts, superficial basal cell carcinomas, actinic keratoses and some case reports as treatment of MEH ([15, 3, 4, 16–20]). In MEH, its use has been primarily described in pediatric and adolescent populations, with one reported case in an adult patient with HIV infection [18]. In all reported cases, a 5% imiquimod has been used with an average application frequency of three times per week over a period of approximately 16 weeks. In three case reports, imiquimod was not employed as a first‐line treatment but rather as part of a combined therapeutic approach, following surgical excision and trichloroacetic acid application, due to inadequate response to initial treatments. Recurrence of MEH lesions using imiquimod has been reported [15]. In contrast, in the present clinical case, imiquimod was administered daily. Complete resolution was observed in cutaneous lesions; however, no significant clinical improvement was noted in mucosal lesions.

Since 2010, the use of trichloroacetic acid in cases of MEH has been described in the literature ([8, 9, 21–24]) and its application has been reported in the pediatric population, ranging from 3 years of age to adolescents. In the literature review conducted, it was demonstrated that trichloroacetic acid has been used at concentrations ranging from 80% to 90%. The application interval for trichloroacetic acid varied among the reports; however, most describe early clinical improvement following its use, with many cases achieving complete resolution of the lesions. In contrast, in the present case, no significant or clinically perceptible improvement has been observed after 1 year of treatment, similar to [22], which documented partial resolution after 5 months of treatment.

The patient was monitored for 27 months, with topical therapies—including 5% imiquimod and 80% trichloroacetic acid—failing to elicit significant lesion regression. The emergence of new lesions was observed. This limited therapeutic response may be attributed to local environmental factors within the oral cavity, such as continuous exposure to saliva, which can dilute and facilitate the clearance of topical agents, thereby reducing their effective concentration and contact time with the lesion. Additionally, mechanical friction from the tongue and other oral structures may further hinder drug retention at the application site, ultimately compromising treatment efficacy. [25].

In the literature, pharmacological treatment of adult patients with persistent MEH remains limited. Consequently, further research is required to evaluate treatment effectiveness in adult populations with this condition.

## Author Contributions

**Daniela Lourdes Argueta-Constanza:** conceptualization, data curation, formal analysis, methodology, resources, validation, visualization, writing – original draft preparation, writing – review and editing. **Lisset Margarita López-Serrano:** conceptualization, data curation, formal analysis, methodology, resources, supervision, validation, visualization, writing – original draft preparation, writing – review and editing. **Florence Juana María Cuadra-Zelaya:** conceptualization, data curation, formal analysis, methodology, resources, supervision, validation, visualization, writing – original draft preparation, writing – review and editing.

## Funding

No funding was received for this manuscript.

## Ethics Statement

All the procedures herein reported were in accordance with the ethical standards of the institutional and/or national research committee involved and the 1964 Helsinki Declaration and its later amendments or comparable ethical standards.

## Consent

The patient′s informed consent was signed.

## Conflicts of Interest

The authors declare no conflicts of interest.

## Data Availability

The data that support the findings of this study are available from the corresponding author upon reasonable request.
